# Aminoglycoside resistance genes *sgm* and *kgmB* protect bacterial but not yeast small ribosomal subunits in vitro despite high conservation of the rRNA A-site

**DOI:** 10.1016/j.resmic.2008.09.006

**Published:** 2008-11

**Authors:** Tatjana Ilic Tomic, Ivana Moric, Graeme L. Conn, Branka Vasiljevic

**Affiliations:** a Institute of Molecular Genetics and Genetic Engineering, Vojvode Stepe 444a, P.O. Box 23, 11010 Beograd, Serbia; b Manchester Interdisciplinary Biocentre, Faculty of Life Sciences, The University of Manchester, 131 Princess Street, Manchester, M1 7DN, UK

**Keywords:** Aminoglycoside resistance, 16S rRNA methylases, Site of action, G1405, Expression in yeast

## Abstract

The aminoglycoside resistance genes *sgm* from *Micromonospora zionensis* and *kgmB* from *Streptomyces tenebrarius* were cloned into a yeast expression vector to test whether the encoded prokaryotic methylases can modify the 18S rRNA A-site and thus confer resistance to G-418. Despite the detectable presence of mRNAs in yeast cells, neither G-418-resistant yeast transformants nor positive western blot signals were obtained. Neither methylase was capable of methylating 40S subunits despite very high conservation of the antibiotic rRNA binding sites. However, the results provide novel insight into the action of Sgm by showing that it methylates the same site as KgmB, i.e. G1405 in 16S rRNA.

## 1 Introduction

The nebramycin antibiotic complex producer *Streptomyces tenebrarius* and the G-52 producer *Micromonospora zionensis* accomplish resistance to their own toxic products through the activity of KgmB and Sgm methylases, respectively [7,14]. KgmB methylates G1405 in the conserved 3′-end of 16S rRNA [1], while the precise methylation site of Sgm is not yet determined. Both methylase genes are negatively autoregulated at the translational level, indicating that only a small number of molecules are needed in prokaryotic cells for effective resistance [8,15].

**Fig. 1:**
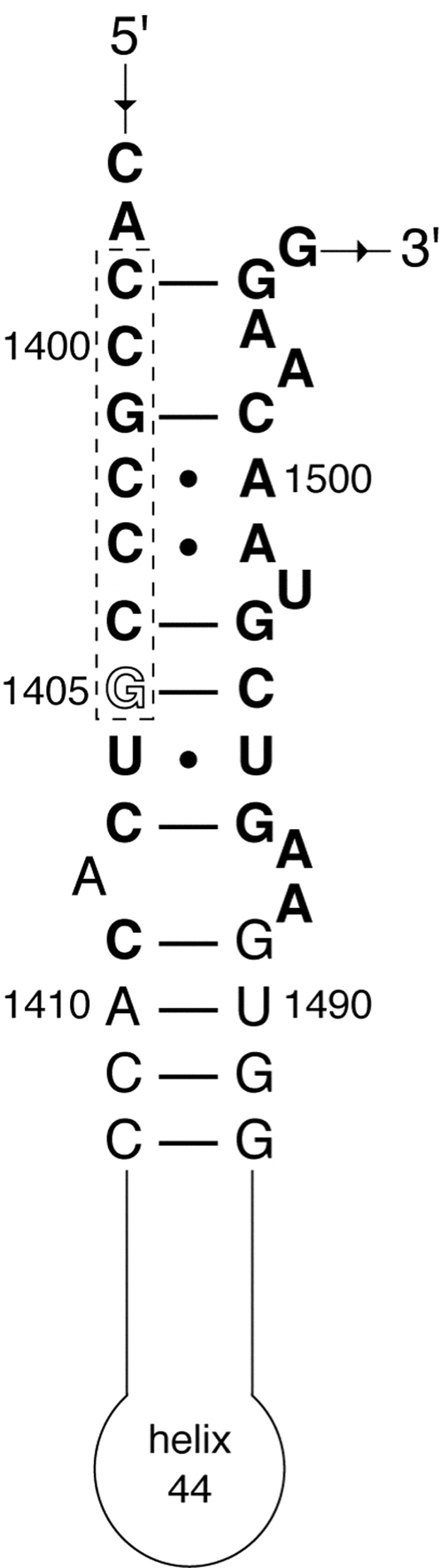
Ribosomal A-site RNA sequence and secondary structure. Residues in bold are conserved between *E. coli* and *S. cerevisiae* ribosomal RNAs. The proposed recognition sequence is boxed with the KgmB and Sgm methylation site in outline font (1405; *E. coli* numbering).

Sequence analysis of bacterial 16S and yeast 18S rRNAs showed high similarity in the aminoglycoside binding region (Fig. 1). Although there is more than 95% identity in the aminoglycoside binding site between rRNAs, aminoglycoside antibiotics do not act on eukaryotic ribosomes, with the exception of G-418. G-418 can bind to the eukaryotic rRNA A-site due to the presence of a 6′-OH group in the ring I and, more importantly, it has ability to induce base-destacking upon binding [5]. This suggested that *sgm* and *kgmB* from *Actinomycetes* might confer resistance to G-418 in the baker's yeast *Saccharomyces cerevisiae* and could potentially be used as a dominant marker in yeast vectors.

Expression of *sgm* and *kgmB* genes and their methylation activities were therefore studied in order to gain new insight into functional interactions of bacterial methylases with prokaryotic and eukaryotic ribosomes.

## 2 Materials and methods

### 2.1 Strains, culture conditions, DNA and RNA techniques

Bacterial transformation (*Escherichia coli* DH5α and MRE600 strains), plasmid preparation, restriction enzyme digestions, ligations and agarose gel electrophoresis were performed as described by Sambrook et al. [13]. For expression of methylase genes in eukaryotic cells, *S. cerevisiae* strain FAS-20 was used [2]. The yeast cells were grown in standard rich nutrient media, YPD or YPGly. The transformation of yeast with different recombinant plasmids was performed as described previously [10]. For isolation of total RNA using a KingFisher^®^ mL kit (Thermo LabSystems), yeast transformants containing pVTS and pVTK expression plasmids were grown in YPD medium until reaching OD_600_ value of 1.0 (24 h).

### 2.2 Plasmid construction

**Fig. 2:**
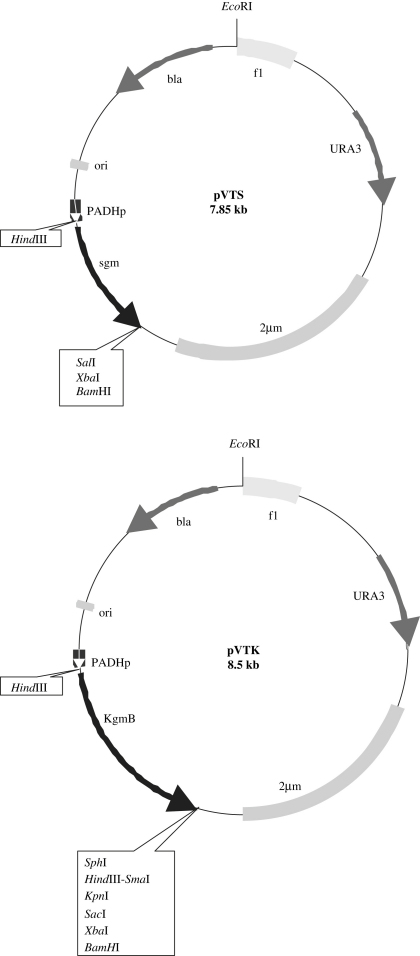
Plasmid maps of pVTS and pVTK. Important restriction sites and the orientation of the reading frames are indicated.

The multicopy yeast shuttle plasmid pVTS was constructed by insertion of a 950 bp *Sma*I–*Bam*HI fragment containing *sgm* into plasmid pVT100-U [16] linearized with *Pvu*II and *Bam*HI restriction enzymes. Plasmid pVTK was obtained by insertion of the 1.6 kb *Sma*I–*Sac*I fragment carrying *kgmB* into pVT100-U digested with *Pvu*II and *Sac*I restriction enzymes. Cloned genes were under control of the constitutive yeast promoter ADHp (Fig. 2.).

### 2.3 Gene expression analysis by reverse transcription PCR (RT-PCR)

Primers used for reverse transcription and PCR amplification of the *sgm* mRNA were antisense: 5′-GCGGCAGGAAGGCGCCG-3′ and sense: 5′-ACCCGGGAAGAGAATGACGGCACCTGCGGC-3′. The *kgmB* mRNA was analyzed in RT-PCR using antisense: 5′-GGAGGGCGTCGTACTTGGG-3′ and sense: 5′-AAGGATCCGCGAGCGGAGAGGACCC-3′ primers. RT-PCR amplification (10 ng of total RNA) was performed using the OneStep RT-PCR kit (Qiagen). After reverse transcription at 50 °C (30 min), reactions were heated at 95 °C for 15 min to simultaneously activate HotStarTaq DNA polymerase and inactivate the reverse transcriptase. PCR amplification was performed in 30 cycles of 94 °C (1 min), 55 °C (1 min) and 72 °C (1 min), with a final extension step at 72 °C (10 min), resulting in approximately 200 bp long products.

### 2.4 Protein extraction and western blot analysis

*S. cerevisiae* cells were grown at 30 °C in either YPD medium or liquid minimal medium (0.7% yeast nitrogen base, 2% glucose and appropriate amino acids) to OD_600_ of 1.5. The cells were harvested by centrifugation and the pellet was resuspended in one of the following buffers:

1. 20 mM Tris–HCl, pH 7.9, 10 mM MgCl_2_, 1 mM EDTA, 5% glycerol, 1 mM DTT, 0.3 mM ammonium sulfate, 1× protease inhibitor mix, 1 mM PMSF. The 100× protease inhibitor mix contained 10 mM benzamidine, 200 μg/ml aprotinin, 50 μg/ml leupeptin, and 100 μg/ml pepstatin A.
2. Ice-cold 50 mM Tris–HCl, pH 7.5, 10 mM NaN_3_ followed by centrifugation, resuspension in 30 μl of 2% SDS, 80 mM Tris–HCl. pH 6.8, 10% glycerol, 1.5% DTT, 0.1 mg/ml bromphenol blue, and rapid heating to 100 °C for 3 min to inactivate proteases.
3. 10 mM phosphate buffer pH 7.2, 5 mM EDTA, 0.5 M NaCl, 0.1% Triton X-100, 1 mM PMSF, 0.1% CHAPS.

The cells were broken by intensive vortexing with glass beads (0.45 μm).

Alternatively, the pellet was resuspended in 100 μl distilled water, 100 μl 0.2 M NaOH was added and incubated for 5 min at room temperature. The cells were then pelleted, resuspended in 50 μl SDS-PAGE sample buffer, boiled for 3 min and pelleted again.

Proteins were separated by SDS-polyacrylamide gel electrophoresis and transferred to a nitrocellulose membrane using Semi-Dry Multiphor II (Pharmacia) for 1 h at 14 mA. The membrane was blocked with 2% non-fat dried milk in washing buffer (10 mM Tris–HCl, pH 8.0, 150 mM NaCl, 0.05% Tween20) and then subjected to immunoreaction with polyclonal antiserum raised against Sgm protein (dilution 500×). The secondary antibody (goat anti-rabbit immunoglobulin G) conjugated with alkaline phosphatase was used as 1:8,000 dilution (Sigma). Immunoblots were developed with 5-bromo-4-chloro-3-indolyl phosphate/nitro blue tetrazolium (BCIP/NBT) as a color substrate according to the manufacturer's instructions (Promega).

### 2.5 Methylation assay of ribosomal subunits

Methylation of ribosomal subunits by 200 pmol of either histidine-tagged Sgm [4] or histidine-tagged KgmB protein [9] was carried out as described previously [7]. Sensitive 30S subunits were isolated from *E. coli* DH5α, resistant 30S subunits from *E. coli* NM522 carrying the *sgm* expression plasmid pQES5 [4] and 40S subunits from *S. cerevisiae* FAS-20 as well as 18S rRNA.

## 3 Results and discussion

### 3.1 Cloning and expression of methylase genes in yeast

To study intracellular expression of *sgm* and *kgmB* in a eukaryotic model system, the yeast/*E. coli* multicopy vector pVT100-U was used [16] and resulting plasmids pVTS and pVTK (Fig. 2) were introduced into *S. cerevisiae* strain FAS-20. These vectors contain a constitutive promoter and the 3′-end of the yeast gene ADHp to ensure expression of cloned genes.

The expression of bacterial methylase genes and consequent yeast resistance to aminoglycoside antibiotic G-418 provided by methylation of 18S rRNA were anticipated based on >95% identity of bacterial and yeast A-sites.

In order to select transformants, two factors were taken into consideration: composition of the growth media, i.e. carbon source, and time of application of selective pressure. Yeast cells with pVTS and pVTK, as well as negative control strains (*S. cerevisiae* strain FAS-20 with or without pVT100-U) were grown in rich YPD or YPGly media, where the carbon source was glucose or glycerol, respectively. As both cytoplasmic and mitochondrial ribosomes are potential targets for antibiotics in yeast, and since cloned resistance genes do not have signal sequences necessary for transport into mitochondria, we exploited the differential effects of fermentative growth on glucose and respiratory growth on glycerol [17]. In the presence of antibiotic, the former regime would determine whether methylation of 18S rRNA occurs. The latter regime, respiratory growth on glycerol (YPGly), was used as negative control in the presence of antibiotic as functional mitochondria are required to produce energy. After reaching either mid-log phase or complete saturation aliquots were used to inoculate fresh liquid media or plated onto solid plates (YPD or YPGly) with increasing concentration of G-418 (10, 20, 30, 50, 100 μg/ml). Different conditions of growth and selection did not result in selection of resistant transformants, indicating that *sgm* and *kgmB* do not provide resistant phenotypes to yeast cells.

### 3.2 Analysis of transcription by RT-PCR

In order to determine if transcriptional failure in expression of Sgm and KgmB took place, reverse transcription (RT)-PCR experiments were performed. The expected PCR products of approximately 200 bp were detected for both genes, indicating that transcription does occur (data not shown).

### 3.3 Analysis of translation by western blot

To clarify whether translational malfunction is the cause of methylase absence in yeast, western blot experiments with rabbit polyclonal anti-Sgm antibodies were performed. Cross-reaction with KgmB (54% identity with Sgm) was previously confirmed [4,9]. Western blot analysis of cell extracts prepared from *S. cerevisiae* transformed with pVTS and pVTK vectors did not produce an expected signal (data not shown) despite extensive variation of both yeast growth conditions and protein isolation protocols, leading us to conclude that Sgm and KgmB were not translated in yeast cells in sufficient amounts to be detected or at all.

Comparison of codon usage in *S. cerevisiae* (www.kazusa.or.jp) with that in *sgm* and *kgmB* revealed that these genes were abundant in codons with low frequency in yeast. As these genes were expressed in yeast from a strong ADHp promoter, the probability of translation of the *sgm* and *kgmB* genes in *S. cerevisiae* is low, though not impossible [6].

### 3.4 Methylation assays

**Fig. 3:**
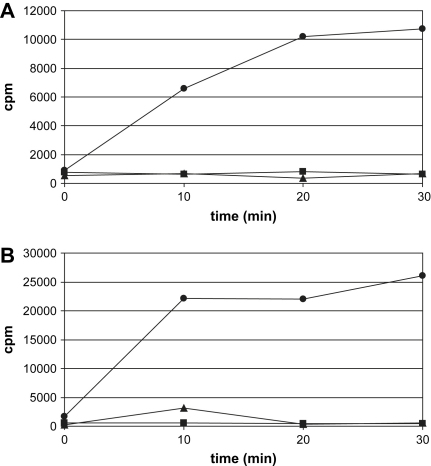
In vitro methylation assays of 30S and 40S ribosomal subunits. Time-course assays for Sgm (A) and KgmB (B) acting on 30S isolated from sensitive *E. coli* strain (circles), 30S isolated from resistant *E. coli* strain (squares) and yeast 40S (triangles). One experiment representative of three repeats is shown.

The capacity of purified proteins to methylate bacterial 16S and yeast 18S rRNA target sequences encompassed by ribosomal small subunits was analyzed using in vitro methylation assays (Fig. 3). Although both methylases could methylate bacterial 30S subunits, neither was able to transfer the methyl group to yeast 40S subunits or naked 18S rRNA (data not shown). The absence of additional methylation by KgmB of in vivo protected subunits isolated from *sgm-*resistant *E. coli* strain strongly suggests that the two enzymes share the same site of action, i.e. G1405.

Since it has been recently shown that members of the KsgA/Dim1 methylase family and their resultant modification of small subunit rRNA are found throughout evolution and that KsgA orthologs from archaeabacteria and eukaryotes are able to complement for KsgA function in bacteria [11], the aim of this work was to test if prokaryotic genes could modify target sequence in 18S rRNA and thus give high levels of aminoglycoside antibiotic resistance.

Despite all similarities in the antibiotic binding site (Fig. 1), methylation by Sgm and KgmB did not occur in yeast, suggesting that the recognition site for these methylases could be different in 30S and 40S subunits. However, footprinting studies have indicated that the A-site region of the 16S rRNA is essentially free of contacts with ribosomal proteins [12]. The different architecture of 40S ribosomal subunit compared to 30S, i.e. proteins adjacent to A-site, is the most likely explanation for inability of Sgm and KgmB methylases to recognize their target sequence. Resistance methylations that have been studied in detail on 16S rRNA tend to occur after assembly of the 30S subunit [3]. This indicates that target nucleotides need to be presented in the higher-order structures that are absent in free rRNA.

The specific molecular recognition events that distinguish the bacterial and yeast ribosome small subunits must be addressed by further structural analysis of Sgm and KgmB and interactions with their targets.
